# Adolescent Tibial Tubercle Fracture: Review of Outcomes and Complications

**DOI:** 10.1007/s12178-023-09849-9

**Published:** 2023-07-12

**Authors:** Chloe Delos Reyes, Wei Wu, Nirav K. Pandya

**Affiliations:** 1 John A. Burns School of Medicine, University of Hawaii, Honolulu, USA; 2 Department of Orthopedic Surgery, Benioff Children’s Hospital, University of California, San Francisco, San Francisco, CA 94609 USA

**Keywords:** Tibial tubercle fracture, Tibial tubercle avulsion, Pediatric knee functional outcome, Pediatric knee complications

## Abstract

**Purpose of Review:**

Fractures of the tibial tubercle are a relatively uncommon injury, representing 3% of all proximal tibia fractures and < 1% of all physeal fractures, primarily seen in the adolescent demographic. While recognition of the injury and its management is being more widely reported in the literature and recognized in the hospital setting, reports of its outcomes and complications have still been limited. This article provides an updated review of the outcomes and complications of tibial tubercle fractures.

**Recent Findings:**

Current research shows both radiographic outcomes, specifically osseous union, and functional outcomes, such as return to play and full knee range of motion, are excellent in patients treated either operatively or nonoperatively. Complication rates overall remain relatively low, with the most common complication being bursitis and hardware prominence and the most common associated injuries being patellar tendon avulsions and meniscus tears.

**Summary:**

With appropriate management, tibial tubercle fractures have an excellent overall outcome and a low complication rate. Although complications are uncommon, treating providers should be vigilant and recognize the signs of devastating complications resulting from acute vascular injuries or compartment syndrome. Further research should aim to analyze patients’ experiences and satisfaction following treatment of this injury and examine the long-term functional and patient-reported outcomes.

## Introduction 

### Background

Fractures of the tibial tubercle represent 3% of all proximal tibia fractures and < 1% of all physeal fractures in the adolescent demographic with increasing incidence, likely a consequence of increased participation in youth sports [1–3]. The tibial tubercle, during this period of bone development of around 14 years of age in girls and 16 years of age in boys, remains susceptible to avulsion throughout the adolescent years as the epiphyseal ossification center begins to fuse with the tibial tubercle ossification center [4, 5]. While generally uncommon, this injury typically occurs in those participating in sports involving forceful contraction of the quadriceps against resistance or with rapid knee flexion, characteristic of jumping and running motions [3, 4]. Because of this mechanism of injury, youth athletes participating in sports such as basketball largely contribute to the demographic of patients who suffer from tibial tubercle avulsion fractures.

Risk factors for tibial tubercle fractures include Osgood-Schlatter disease, a condition in which stress on the patellar tendon, commonly seen with repetitive loading on the tibial tubercle, results in pain and swelling over the tibial tubercle with subsequent tubercle prominence. Osteogenesis imperfecta, an inherited disorder that affects the density and integrity of bone, may also be a predisposing factor to tibial tubercle fractures. However, reports of such complications in patients with either Osgood-Schlatter disease or osteogenesis imperfecta are rare [6, 7]. Additionally, extreme body mass index (BMI), defined as being in the 5th percentile or in the 97th percentile, may be a risk factor for tibial tubercle avulsion fractures in adolescents without definite trauma, possibly due to abnormal loading on a weak physis in obese children or low bone strength related to bone mineral density in underweight children [3].

Tibial tuberosity fractures may be classified radiographically by the Ogden, Watson-Jones, Ryu and Debenham, and Pandya classification systems. The Watson-Jones system was the first to classify tibial tubercle fractures, categorizing the fracture pattern into one of three types depending on location and involvement. Type I represents a fracture of the distal tibial tubercle, type II involves the physis while sparing the knee joint, and type III involves extension into the joint space [8]. The Ogden classification, which is the most commonly used system, expands upon the Watson-Jones system with modifiers “A” and “B” to indicate a displaced or comminuted fracture, respectively [9]. Ryu and Debenham further expanded upon the Watson-Jones system by including a type IV categorization which is marked by an avulsion fracture of the entire proximal tibial epiphysis [10]. Most recently, Pandya proposed a new four-tier classification system that takes into consideration the complex, three-dimensional anatomy of the fracture and pattern of proximal tibial physeal closure [11]. In this system, type A fractures are classified as an isolated fracture with an ossified tip, type B involves an epiphysis and tubercle fracture from the metaphysis without intra-articular involvement, type C fractures have intra-articular involvement and extend into the proximal tibia, and lastly, type D fractures involve the distal tubercle [4]. Such a classification system aims to guide the use of advanced imaging and additional surgery to more promptly address potential associated injuries that may have been overlooked in radiographic analysis.

Fracture classification has long dictated the treatment of tibial tubercle fractures, which include nonoperative management for minimally displaced fractures with or without closed reduction, or operative treatment through open reduction and internal fixation (ORIF) with or without arthroscopy for fractures with unacceptable displacement > 2 mm, need for an intra-articular reduction, or soft tissue repair. However, a recent study suggests the choice of treatment may depend more on the degree of reduction immediately achieved in the emergency room and the patient’s ability to extend the knee over the fracture type, which may guide future treatment of tibial tubercle fractures and limit the need for surgical intervention [11].

### Purpose of Current Review

While both radiographic and functional outcomes following fracture of the tibial tuberosity have been reported to be excellent in the majority of case series, potential sequelae include soft tissue damage, compartment syndrome, meniscus damage, and disruption of the patellar tendon [4, 12–15]. Complications following tibial tubercle fractures are rare overall; however, it is important to recognize these complications nonetheless. Given the rarity of this injury, with most studies limited to case series, information regarding the outcomes and complications remains relatively limited. Therefore, the purpose of this study is to expand on current research and to provide an updated comprehensive review of the outcomes and complications of tibial tubercle fractures seen in the adolescent population.

## Outcomes of Tibial Tubercle Fractures

### Methods of Treatment

The primary objectives in the management of tibial tubercle fractures are to restore the knee extensor mechanism and articular surface of the tibia and to address other associated injuries if present [11, 16, 17]. While treatment options include both open and closed fixation methods, fixation technique is largely based on surgeon comfort, and the presence of other injuries, such as disruption of the patellar tendon, menisci, or cruciate ligaments, should be considered when determining treatment modality [18•].

A large retrospective case series by Haber et al. reported that nonsurgical treatment was more commonly performed in females, type I fractures, patients with pre-existing Osgood-Schlatter disease, and patients with body mass index (BMI) ≤ 20 [19••]. Nonoperative treatment may be carried out with or without closed reduction. Closed reduction involves immobilization in a long leg or cylinder cast above the proximal patella for approximately 4 weeks or until the union is evident on radiographs. Such treatment is primarily indicated for Ogden types IA, IB, and IIA fractures in which the fracture is non-displaced or minimally displaced [11, 17]. However, a recent case series by Checa et al. followed five patients, including three cases of type IV fractures, whose treatment consisted of split immobilization in full extension in the emergency room or operating room, depending on the level of pain. All patients had excellent outcomes, with all returning to play in fewer than 25 weeks, suggesting early anatomical reduction may serve as a better guide for treatment rather than the fracture classification [20].

A systematic review by Pretell-Mazzini et al. reported up to 88% of cases of tibial tubercle fractures are candidates for surgery, with open reduction and internal fixation (ORIF) accounting for 98% of surgical correction [12]. ORIF is largely considered the standard of care, especially for patients whose injuries involve epiphyseal or intra-articular extension, which include Ogden types IIB, IIIA, IIIB, and IV [13, 17]. The procedure involves fixation with screws, washers, tension band wiring, or suture repair of the periosteum, followed by casting for 3–4 weeks [16, 17]. If electing to under ORIF, joint exploration and treatment of soft tissue injuries, such as avulsions of the quadriceps or patellar tendon, may also be pursued [17, 18•]. Arthroscopic techniques can also be used to help reduce the incision size, need for large arthrotomy, and to assess the adequacy of cartilage restoration [16, 18•, 19••].

### Radiographic Outcome

A review of the literature suggests the radiographic outcomes following fractures of the tibial tubercle treated either operatively or nonoperatively have been good, with almost all cases eventually achieving osseous union. Fracture union has been reported in up to 99.8% of cases, regardless of treatment modality [12, 21••]. For patients with non-displaced fractures treated nonoperatively, full union and functional recovery has been reported in the literature [22]. A retrospective review by Pace et al. looked at 130 operative tibial tubercle apophyseal fractures and found a 100% radiographic union rate in 18 patients with type IV fractures treated operatively [23]. In Jardaly et al.’s retrospective review comparing surgical outcomes between patients treated via ORIF and closed reduction and internal fixation (CRIF), they found that all 17 CRIF cases healed to the union, as did 80 out of 81 ORIF cases [18•]. A summary of these findings can be found in Table 1.

**Table 1:** Summary of radiographic outcomes following management of tibial tubercle fractures

Study	Incidence
Pretell-Mazzini et al. [12]	334 of 336 (99%)
Kalifis et al. [21••]	954 of 956 (99.8%)
Pace et al. [23]	24 of 24 (100%)
Jardaly et al. [18•]	97 of 98 (99%)

Reported incidence of osseous union achieved in patients whose fractures were treated both operatively and nonoperatively

### Functional Outcome

Functional outcomes following treatment of tibial tubercle fractures have been reported to be excellent in over 95% of cases treated operatively, with up to an average of 98.9% returning to play at an average of 3.9 months post-operatively and 98% achieving a full knee range of motion at an average of 22 weeks post-operatively [12, 14–16, 21••, 24, 25]. When comparing terminal extension, terminal flexion, and total arc of motion between injured and uninjured extremities at follow-up, there were no significant differences [13]. However, in a retrospective case series of 228 patients with 236 tibial tubercle fractures examined by Haber et al., 40 patients reported residual pain on palpation of the tibial tubercle or surgical site, while 23 patients reported pain with squatting [19••]. The authors also reported limited acute flexion and extension in 1% of fractures [19••]. A summary of these findings can be found in Table 2.

**Table 2:** Summary of functional outcomes following management of tibial tubercle fractures

Parameter	Incidence	Study
Residual pain on palpation	40 of 228 (18%)	Haber et al. [19••]
Pain with squatting	23 of 228 (10%)	Haber et al. [19••]
Full knee ROM	250 of 255 (98%)	Pretell-Mazzini et al. [12]
Return to play	248 of 264 (94%)	Pretell-Mazzini et al. [12]
	99 of 112 (88%)	Haber et al. [19••]

*ROM*, range of motion

Reported incidence of functional outcomes at final follow-up

## Complications of Tibial Tubercle Fracture and its Treatment

### Osseous Complications

Nonunion and refracture of the tibial tubercle are rare complications following treatment, as the rate of osseous union is reported to be as high as 99.8% of cases. However, Haber et al. reported nonunion, displacement, or growth disturbance occurring in less than 3% of cases [19••]. Additionally, in a systematic review by Pretell-Mazzini et al., refracture was seen in approximately 6% of patients [12].

### Neurovascular Complications

Acute compartment syndrome poses a very concerning complication that is thought to be the result of disruption of branches from the anterior recurrent tibial artery which traverses near the lateral border of the tibial tubercle, rendering it susceptible to injury upon fracture of the tibial tubercle [4, 12]. In a retrospective review of 19 adolescents with 20 tibial tubercle fractures by Frey et al., 4 patients, which included type IIA, IIB, and IV fractures, presented with compartment syndrome, all of whom required fasciotomies [16]. The presentation of compartment syndrome may differ in the pediatric population, with potential signs including increased narcotic requirement, increased anxiety, and restlessness; therefore, vigilance must be practiced by the healthcare team when managing these injuries [26]. In Haber et al.’s review, iatrogenic injury to the popliteal artery injury was reported in one case due to drilling from anterior to posterior [19••]. Lastly, in a review of 90 fractures in 86 patients treated surgically by Arkader et al., postoperative numbness over the tubercle was reported by two patients [27].

### Physeal Complications

Physeal complications and growth disturbances remain a rare and infrequent complication as the physis nears normal closure around or after the time most fractures of the tibial tubercle are seen between ages 12 and 17 [22, 28]. However, there has been one reported case of premature closure of the proximal tibial physis in a patient with myelomeningocele, resulting in leg length discrepancy [9]. In a systematic review conducted by Pretell-Mazzini et al., genu recurvatum was reported in 4 out of 95 cases [12]. Haber et al. also reported two cases that resulted in a 1–2 cm limb length discrepancy that subsequently, although uncommon, resulted in genu recurvatum [19••].

### Hardware-associated Complications

The most reported hardware-associated postoperative complication is tenderness or prominence over the tibial tubercle. In Haber et al.’s review, 39 patients reported hardware prominence or irritation prior to hardware removal [19••]. Pretell-Mazzini et al. reported an overall 28% complication rate (95 of 336 fractures), with bursitis accounting for 56% of the complications (53 of 95), requiring subsequent removal of the implants [12].

### Stiffness

Stiffness is not a commonly reported complication; however, it was reported in one case by Frey et al. examining 19 patients with 20 tibial tubercle fractures treated with ORIF [16]. In Pretell-Mazzini et al.’s review, 3 patients reported stiffness as a complication [12].

### Infection

In Haber et al.’s retrospective case series of 124 cases, 88 of which were surgical, acute surgical complications included 6 patients experienced postoperative infections or wound complications, and 5 patients required oral antibiotic treatment for superficial infection [19••]. Wound infections specifically have also been reported by Pretell-Mazzini et al. in 3 cases, all of which were superficial [12].

### Associated Injuries

Kalifis et al. found that from the 25 retrospective cohort studies they examined, there were 919 patients with 956 tibial tubercle fractures in which associated injuries were reported in approximately 10% of cases [21••]. Although other injuries associated with tibial tubercle fractures are generally rare, the most common associated injury has been patellar tendon avulsions, with partial and/or complete ruptures seen in approximately 2 to 15% of cases [11, 14, 18•]. Meniscal tears follow behind patellar tendon avulsions in incidence and often occur with intra-articular involvement [11]. Other injuries that have been reported include soft tissue damage, periosteal damage, avulsion of the distal patella, coronary ligament damage, articular cartilage damage, and joint laxity [27, 29, 30]. Cole et al. also reported skin tenting as a possible injury leading to necrosis of the skin which would warrant immediate surgery [31•]. Table 3 provides a summary of all the reported complications.

**Table 3:** Summary of complications following management of tibial tubercle fractures

Complication	Incidence	Study
Osseous		
Nonunion	1 of 99 (1%)	Haber et al. [19••]
Refracture	6 of 95 (6%)	Pretell-Mazzini et al. [12]
Neurovascular		
Acute compartment syndrome	4 of 19 (21%) 4 of 228 (2%)	Frey et al. [16] Haber et al. [19••]
Iatrogenic popliteal artery injury	1 of 88 (1%)	Haber et al. [19••]
Numbness	2 of 86 (2%)	Arkader et al. [27]
Physeal		
Leg length discrepancy	2 of 99 (2%)	Haber et al. [19••]
Genu recurvatum	4 of 95 (4%)	Pretell-Mazzini et al. [12]
Hardware-associated		
Hardware prominence	39 of 99 (39%)	Haber et al. [19••]
Bursitis	53 of 95 (56%)	Pretell-Mazzini et al. [12]
Stiffness	1 of 19 (5%) 3 of 95 (3%)	Frey et al. [16] Pretell-Mazzini et al. [12]
Infection		
Postoperative or wound	6 of 88 (7%)	Haber et al. [19••]
Superficial	3 of 95 (3%)	Pretell-Mazzini et al. [12]
Associated injuries		
Patellar tendon avulsion	10 of 98 (10%) 21 of 228 (9%)	Jardaly et al. [18•] Haber et al. [19••]
Meniscus tear	6 of 336 (2%)	Pretell-Mazzini et al. [12]

## Conclusion

The overall outcome of tibial tubercle fractures is good with appropriate treatment, and the overall complication rate is low. Based on current literature, devastating complications are rare. However, clinicians should stay vigilant to rule out acute vascular injuries or compartment syndrome. While data is limited, there is a need for long-term follow-up on functional and patient-reported outcomes and on patient experience and satisfaction after treatment of this injury.
